# Editorial: The intersection among the internet and social media use, mental health, and Trust & Safety practices

**DOI:** 10.3389/fpsyt.2023.1332930

**Published:** 2023-11-20

**Authors:** Xieyining Huang, Chiara Massullo, Larisa T. McLoughlin

**Affiliations:** ^1^TaskUs Inc., New Braunfels, TX, United States; ^2^Department of Education, Roma Tre University, Rome, Italy; ^3^Department of Mental Health and Suicide Prevention, Wellbeing SA, Adelaide, SA, Australia

**Keywords:** social media, mental health, Trust and Safety, content moderation, internet

## Vision

In our increasingly digital world, the presence of online platforms has become an undeniable reality. Whether we welcome this transformation or not, it is nearly impossible to ignore the profound impact these platforms have on our daily lives. Discourse on understanding such impact appears to coalesce into three main domains: (a) safety of the platform users; (b) Trust and Safety (T&S) practices of the platforms, which may include policies and design choices; and (c) safety of the professionals who enforce platform T&S policies, such as content moderators.

Historically, these domains were largely examined in isolation from one another. A wealth of knowledge exists in academia on the potential impact of online platforms on users' wellbeing (1, 2), but most studies were conducted from a purely academic perspective with few studies originating with a collaboration between academia and industry. Given this dearth of publicly available knowledge that is both theoretically relevant for academia and practical for industry implementation, T&S policies and practices are at times crafted based on well-intentioned but untested assumptions. It is important to note that some individual platforms do conduct critical research within their own organization to inform T&S decisions. However, it is difficult to discern the extent and breadth of this internal knowledge as few studies are published externally. The limited amount of industry research that is shared publicly has faced criticism of non-objectivity and potential bias despite its high relevance for real-world applications. Furthermore, the critical work of content moderators, which is integral to maintaining the safety of online communities, has historically received scant attention in both academic and industry research. This is a critical gap of knowledge as it is challenging—if not impossible—to enforce T&S policies and ensure the safety of online users without ensuring the safety of content moderators.

The time has come for us to recognize that these domains cannot and should not be examined in isolation. The challenges of creating and sustaining safe online communities are immense, and they necessitate a collective effort. In recent years, we have witnessed commendable efforts to foster more open and transparent dialogues and collaboration among academia, industry, civil society, and government. Initiatives such as the first journal dedicated to T&S, the establishment of the first Trust & Safety research conference, and the founding of the Trust and Safety Professional Association (TSPA) and Trust and Safety Foundation (TSF) signify the growing recognition of the need for joint efforts in this arena. However, we still have much room to improve.

This is precisely why we have initiated this special Research Topic, “*The intersection among the internet and social media use, mental health, and Trust & Safety practices*,” to serve as yet another channel to bridge the gap and stimulate open dialogues. We aspire to use this opportunity to bring together academic researchers and industry entities with the shared goal of making online platforms safer for everyone.

## Special topic research perspectives

In this special topic collection, a total of four empirical research articles tackle a range of issues and represent perspectives from academic and industry organizations across the globe. First, academic researchers (Piksa et al.) from Norway and Poland conducted a study on misinformation and found that users were more likely to engage with a news item when it aligns with their preconceived beliefs. This study adds to the existing literature on the spread of misinformation on online platforms and suggests that traditional fact-checking interventions may be insufficient to reduce misinformation.

Second, academic researchers (Son et al.) from Korea found correlations between suicide and self-injury attempts with search term volumes on these topics. This finding supports the potential utility of leveraging online systems to predict suicide risk and offer interventions.

The third paper (Kawabe et al.) studied teacher awareness of internet addiction among elementary school students in Japan. The study showed that most teachers did not approach students with problematic internet use and had little knowledge on how to best intervene. The study called for more education and support for professionals who regularly interact with students so that they can help promote healthy internet use among minors.

Lastly, Torralba et al. established the first trait-based employment screener for content moderators in the Philippines. By assessing traits that may predict success in content moderation, this screener demonstrated the potential to protect thousands of content moderators' psychological health. This study also represents a collaboration between academic and industry institutions; it showcases how impactful research can be when the strength of academic research (e.g., research rigor) is combined with the strength of industry research (e.g., ecological validity, scalability).

Together, these articles highlight the many, though by no means exhaustive, facets of challenges that we must solve to promote a safe and healthy online experience: misinformation, suicide and self-injury, wellbeing for youth and children, and protection of our frontline T&S professionals. The articles contained within this special edition provide us with a glimpse into the immense potential that lies in uniting research on topics that were often studied separately.

## A call for ongoing collaboration and research

This special topic collection signifies the potential for interdisciplinary collaboration and cross-sector engagement in addressing the multifaceted challenges of the digital age. We hope that this issue will inspire further partnerships between academia and industry and serve as a catalyst for even more impactful research that promotes a safer, healthier, and more responsible digital world.

## Author contributions

XH: Writing—original draft, Writing—review & editing. CM: Writing—review & editing. LM: Writing—review & editing.
